# Endoglin: Beyond the Endothelium

**DOI:** 10.3390/biom10020289

**Published:** 2020-02-12

**Authors:** Mark J. A. Schoonderwoerd, Marie-Jose T. H. Goumans, Lukas J. A. C. Hawinkels

**Affiliations:** 1 Department of Gastrenterology-Hepatology, Leiden University Medical Center, 2300 RC Leiden, The Netherlands; 2 Cell and Chemical Biology, Leiden University Medical Center, 2300 RC Leiden, The Netherlands

**Keywords:** endoglin, CD105 TGF-β, BMP9, ALK-1, TRC105, tumor microenvironment

## Abstract

Endoglin, a type-III accessory receptor for the Transforming Growth Factor (TGF)-β superfamily pathway, is known for its crucial role during angiogenesis. Extensive work has shown the important roles that endoglin plays in balancing the TGF-β signaling pathway, thereby regulating endothelial cell proliferation and migration. However, recent work indicates a far more widespread role for endoglin beyond the endothelial cells. In this review, we will provide a summary of recent publications on endoglin expression on epithelial (cancer) cells, cancer-associated fibroblasts, and mesenchymal stem cells. Additionally, we will discuss the role of endoglin in innate and adaptive immunity. Finally, we will discuss the results of clinical trials using the endoglin targeting antibody (TRC105), focusing on the effects observed beyond the endothelium. In conclusion, although endoglin was initially identified as an endothelial marker, additional roles for endoglin on other cell types have been shown, although the number of studies is still limited, with sometimes conflicting data. Future studies will further establish the roles of endoglin beyond the endothelium.

## 1. Introduction

Endoglin is a 180 kDa, type-I transmembrane glycoprotein and functions as a coreceptor for ligands of the Transforming Growth Factor (TGF)-β superfamily. Endoglin is predominantly expressed by activated endothelial cells [1] and plays a crucial role in (developmental) angiogenesis. In mice, a complete loss of endoglin is embryonically lethal around embryonic day 10.5, primarily due to impaired development of the vascular plexus into a mature vascular network, causing hampered low and osmotic imbalance, disturbing normal cardiac development [2,3]. Part of the cardiac abnormality is caused by pericardial effusion due to disturbed osmotic balance [2]. This indicates the pivotal role that endoglin plays in developmental angiogenesis. Early work has shown that endoglin contributes to angiogenesis by regulating the proliferation [4] and migration [5,6,7] of endothelial cells [7]. This work has been extended, with multiple studies showing an important role for endoglin in tumor angiogenesis and strategies for inhibiting tumor angiogenesis by targeting endoglin.

The role of endoglin in developmental and tumor angiogenesis has been extensively reviewed elsewhere [8,9,10,11]. However, more recent studies have reported novel roles for endoglin signaling in (cancer-associated) fibroblasts (CAFs), Mesenchymal Stromal Cells (MSCs), epithelial cancer cells, and various immune cell subpopulations. This review highlights the current knowledge on endoglin expression and function on non-endothelial cells and what implications this might have. 

## 2. Endoglin Structure and Function

Endoglin (CD105) is a homodimeric transmembrane receptor composed of disulphate bond-linked subunits of 95 kDa [12] and is highly homologous between species [13,14]. In humans, the endoglin gene is located on chromosome 9 [15] and is composed of exons 1 to 8, 9A and 9B, and 11 to 14 [16,17]. Endoglin has a short cytoplasmic domain, which reflects its co-receptor function modulating the response, rather than initiating the signaling cascade [18]. Therefore, it requires additional receptors to induce signaling. In both human and mouse tissues, two spliced isoforms—long- (L) and short- (S) endoglin—have been reported [19]. S-endoglin and L-endoglin proteins vary from each other in terms of their cytoplasmic tails, which contain 14 and 47 amino acids, respectively [20,21]. L-endoglin is the predominantly expressed isoform and promotes signaling via the ALK1 pathway, while S-endoglin seems to promote the ALK5 pathway [19]. Activation of the activin receptor-like kinase (ALK)1 and ALK5 pathways leads to the downstream activation of the smad1/5/8 or smad2/3 pathway (see below), respectively, resulting in the transcription of different target genes. In terms of the exact role of S-endoglin, not much is known. It has been reported that transgenic mice with endothelial specific Intercellular Adhesion Molecule 2 (ICAM-2) S-endoglin overexpression show a decreased response to nitric oxide (NO) inhibition, which was associated with a hypertensive response. Furthermore, decreased TGF-β1 responses were detected in these endothelial cells, indicating that the upregulation of S-endoglin is part of the senescent program of endothelial cells [22].

Endothelial endoglin expression is regulated by TGF-β, bone morphogenetic protein (BMP)-9 [23], and hypoxia [24]. A hypoxia responsive element was identified downstream of the endoglin promoter, which can bind the hypoxia-inducible factor (HIF)-1a, resulting in increased endoglin transcription [24]. Furthermore, the stimulation of endothelial cells shows the ligand-dependent upregulation of endoglin expression. Endoglin, however, is not only regulated on the transcriptional level. Cell-surface endoglin expression is also regulated via receptor shedding. Our previous work showed that the membrane-bound protease Matrix Metalloproteinase-14 (MMP-14, also known as Membrane Type-1 MMP) is able to cleave endoglin in the extracellular domain close to the cell membrane [25], and the same phenomenon was seen by Aristorena et al. for MMP-12 secreted by inflammatory macrophages [26], which generated a soluble form of endoglin (sol-eng). Sol-eng can disturb vascular remodeling and maintenance, resulting in vascular abnormalities. High levels of sol-eng have been measured in the circulation of women developing preeclampsia—a disease characterized by a high blood pressure and vascular abnormalities during pregnancy [27]. In cancer, different studies have reported conflicting data about the levels of sol-eng [28,29,30]. The anti-angiogenic function described for sol-eng suggests that it has a tumor-suppressor role in cancer, which is in contrast to studies reporting that high levels of sol-eng correlate to poor patient prognosis [29].

Sol-eng was originally described to inhibit angiogenesis by acting as a ligand trap for the endoglin ligand TGF-β [31] or, as has been more commonly reported, BMP-9 [32,33]. Interestingly, recent data indicate that in addition to being an inhibitory ligand trap, increased circulating monomeric sol-eng might stimulate BMP-9 signaling via binding to endothelial endoglin. The authors have demonstrated that the binding of monomeric sol-eng to BMP-9 does not inhibit BMP-9 signaling in endothelial cells [34], but potentiates it. Furthermore, they have shown that sol-eng in plasma from preeclampsia patients primarily consists of a monomeric sol-eng form, suggesting that sol-eng in this case would not act as an inhibitory ligand trap for BMP9. For cancer, this has yet to be investigated. Taken together, these data show that the role of sol-eng in regulating angiogenesis might be more complex than originally anticipated.

### 2.1. Endoglin Signaling Pathways and Ligands

Members of the TGF-β family exert their cellular effects by binding to a complex of type-I and type-II transmembrane receptors. Seven type-I receptors, also known as ALKs, and five ligand-binding type-II receptors, have been identified [35,36]. Upon ligand binding, a heterotetrameric receptor complex is formed, resulting in the transphosphorylation of the type-I receptor on specific serine and threonine residues in the intracellular region by the constitutively active type-II receptor. Endoglin (a type-III receptor) is reported to play an important role in balancing the TGF-β signal in endothelial cells, by regulating the recruitment of different type-I receptors. Next to the TGFβ type-I receptor ALK5, endoglin can induce TGFβ signaling via ALK1 in endothelial cells [37,38]. Endoglin interacts with TGF-β1 and TGF-β3, but only when it is associated with TGF-βRII. Furthermore, the endoglin extracellular and intracellular domains interact with both TGF-βRII and ALK5 [10]. ALK5 then phosphorylates smad2/3 and translocates to the nucleus, together with the common smad4. In the presence of endoglin, ALK1 is recruited into this complex, shifting the pathway activation towards the ALK1 kinase. Next to TGF-β, endoglin is able to bind BMPs directly via a complex with ALK1. This complex is then able to bind BMP-9 with much higher affinity than to TGF-β1, up on receptor complex activation. ALK1 then phosphorylates smad1/5/8, together with smad4, and this complex translocates to the nucleus, where it increases proliferative signals in endothelial cells [39,40,41]. However, it must be noted that contradicting results on the role of BMP-9 in inducing angiogenesis have been reported, with some also showing a potential inhibitory role for BMP-9 in angiogenesis [23,42]. The contradictions might be due to the use of different cell types, receptors, and concentrations of ligands. 

Next to endoglin, betaglycan is also an accessory type-III receptor for the TGF-β signaling pathway [43]. The main function of betaglycan is presenting ligands to the TGF-β signaling receptors [44]. Betaglycan is important (especially in vivo) for enabling TGFβ2 to exert an effect. TGF-β2 binds poorly to the TGF-β type-II receptor in the absence of betaglycan [45]. Furthermore, betaglycan is crucial during reproduction [46] and fetal development [47], and acts as a potent tumor suppressor in many different types of tumors. [48,49,50]. The interaction between betaglycan and GAIP-interacting protein C-terminus GIPC is required for TGF-β type-III-mediated suppression of the TGF-β signaling and invasion [48].

### 2.2. Endoglin and Developmental/Tumor Angiogenesis

Hereditary hemorrhagic telangiectasia (HHT), also known as Rendu-Osler–Weber syndrome, is a rare genetic disease, which is characterized by mutations in the endoglin (HHT-1) or ALK-1 (HHT-2) [51] genes. HHT-1 is more severe than HHT-2 and is associated with vascular abnormalities in the lungs and brain. HHT occurs in 1 in 5000–8000 people in North America and is found more frequently in people form the Netherlands Antilles (Bonaire and Curaçao). Although patients suffer from frequent nose bleeds and arteriovenous malformations in the brain, lung, and liver, most HHT patients have a normal lifespan. Mechanistic studies on HHT have been performed in endoglin heterozygote mice, which, in contrast to endoglin-knockout mice, are viable and show signs of HHT, such as telangiectasias and nosebleeds, after a prolonged period of time [52]. A complete overview of the models used to study HHT has been carefully reviewed by Tual-Chalot et al. [53]. Endoglin-knockout mice have defects in endothelial cell-dependent smooth muscle cell recruitment [54]. Studies of HHT patients have shown that they display a decreased number of lymphocytes compared to healthy controls [55]. Furthermore, HHT patients have an increased risk of severe bacterial infections due to defects in both polymorphonuclear and monocytic cells [56], stressing the importance of endoglin, as further discussed below. Endoglin heterozygote mice and HHT patients can reveal important information on the role of endoglin, both in and beyond angiogenesis. 

### 2.3. Non-Ligand-Dependent Interactions (Integrins/Leukocyte Trafficking)

As described above, endoglin-dependent signaling can directly influence endothelial cell migration and proliferation in a TGF-β/BMP-9-dependent manner. In addition, several ligand-independent interactions of endoglin have been reported. After the original identification of endoglin, it was already discovered in 1992 that endoglin can bind to integrins on leukocytes [57,58], allowing them to extravasate in a process called Trans Endothelial Migration (TEM). Integrins are ubiquitous cell surface receptors involved in cell–cell and cell–matrix interactions [59]. The functional role of endothelial endoglin as a receptor for integrins on leukocytes has been reported by Rossi et al. [60]. An interesting observation they made was the ability of sol-eng to inhibit leukocyte adhesion to endothelial cells [60], suggesting that sol-eng binds to integrins on leukocytes, thereby blocking their extravasation. 

Although most studies have focused on myeloid cells, T-cells have a major contribution to immune responses during viral infections and anti-tumor immunity. Therefore, it might be of great interest to investigate the endoglin-dependent TEM of T-cells in cancer patients. Taken together, sol-eng might inhibit the TEM of pro-inflammatory cells and/or anti-inflammatory cells, which might be of great interest and a possible therapeutic target. An excellent review on the interaction of endoglin with integrins has been published [61].

### 2.4. Endoglin beyond the Endothelium

As discussed above, endoglin plays a crucial role in angiogenesis and leukocyte trafficking via ligand-dependent and -independent interactions. Endoglin was originally identified in 1985 as a protein expressed on the pre-B leukemia cell line [62]. However, for a long time, research almost exclusively focused on endoglin expression on endothelial cells. More recent work has shown endoglin expression on a variety of other cells, with distinct roles in their behavior. Below, we will discuss the various studies in which non-endothelial endoglin expression has been investigated.

### 2.5. Endoglin Expression on Epithelial Cells 

In normal epithelial cells, endoglin expression has been studied during wound healing [63], where enhanced endoglin expression was found in mouse epidermal keratinocytes. In vivo endoglin was associated with hyperproliferation [64]. Endoglin expression on epithelial cells has been a subject of debate for quite some time. In prostate cancer, the loss of epithelial endoglin expression has been associated with increased metastatic behavior, both in vitro and in vivo, in orthotopic mouse models for prostate cancer [65,66]. In breast cancer, endoglin expression has been investigated in a subset of invasive breast cancer cell lines. The expression of endoglin in MDA-MB-231 cells blocks TGF-β-enhanced cell motility and invasion and reduces lung colonization in a murine metastasis model [67].

Furthermore, in a large breast cancer patient cohort, it was shown that a lack of endoglin expression on tumor cells correlates with a poor clinical outcome [67]. Similar findings have been reported in esophageal squamous cell carcinoma, where a lack of endoglin expression was associated with decreased migration and colony formation in vitro [68]. These data suggest that endoglin might act as a tumor suppressor in both breast cancer and esophageal squamous cell carcinoma. 

In contrast, other reports have described a pro-tumorigenic role for endoglin expression on epithelial cancer cells. For hepatocellular carcinomas (HCC), it has been shown that endoglin expression on HCC cells promotes metastasis in a vascular endothelial growth factor (VEGF)-dependent manner [69]. Next to HCC, endoglin expression in ovarian cancer and renal cell carcinoma has been linked to a stem-cell-like phenotype, accompanied by higher invasion in Transwell migration assays [70]. Furthermore, it has been revealed that endoglin induces epithelial to mesenchymal transition (EMT), but not metastasis, in clear cell renal cell carcinoma [71].

Taken together, the collective data on epithelial endoglin expression have revealed that it is tumor type-specific but the role of endoglin in epithelial cancer cell behavior is not yet fully understood. To elucidate this further, more mechanistic studies, supported by protein expression data in clinical samples, are needed to draw firm conclusions.

### 2.6. Endoglin Expression during Haematopoiesis

**Figure 1:**
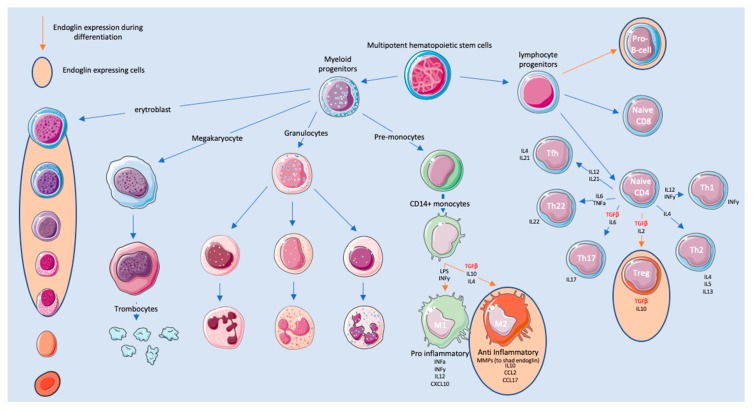
Endoglin during the development and differentiation of immune cells.

Hematopoietic stem cells (HSCs) are constantly replenishing all types of blood cells present in circulation throughout the lifespan of an individual. These new cells are ‘born’ in the red bone marrow in the center of most bones. Endoglin is expressed on long-term repopulating HSCs [72]. Rokhlin and co-workers have shown that endoglin is expressed in maturating erythroblast stromal cells and a subset of CD34+ HSCs. The CD34+ HSCs were eventually found to be B-cell-committed progenitor cells [73]. The overexpression of endoglin in HSCs increased erythroid differentiation during the basophilic erythroblast phase, suggesting its key role in adult erythropoietic development [74]. Furthermore, endoglin expression was also found on monocytes [58]. Finally, endoglin has been demonstrated during the differentiation of HSCs to blood cells (Figure 1). The role of endoglin in innate and adaptive immunity will be discussed in more detail below.

### 2.7. Endoglin Expressed on Innate Immune Cells 

The immune system is divided into innate and adaptive immunity. The innate immune system is composed of several cell types, including neutrophils, eosinophils, basophils, mast cells, and monocytes/macrophages, of which the latter have been reported to express endoglin. Monocytes are derived from hematopoietic stem cells in the bone marrow and spleen [75], before they enter the circulation. Upon entering the blood stream, two subsets of monocytes can be distinguished. One subset is recruited into the tissue throughout the entire body [76], while the second subset has endothelial cell-supporting functions [77]. During monocyte differentiation, endoglin is highly expressed [58,78]. Interestingly, endoglin seems to be involved in the differentiation of monocytes into both M1 and M2 macrophages in the tissue (Figure 1).

M1 macrophages are characterized by their pro-inflammatory and anti-tumor functions and secretion of inflammatory cytokines, whereas M2 macrophages are known for their anti-inflammatory and pro-tumor functions. Furthermore, M2 macrophages are characterized by the expression of c-myc [79]. Little is known about the regulation and function of endoglin on these cells [80]. Endoglin expression on M2 macrophages leads to the downregulation of c-myc, which implies that endoglin might be responsible for the polarization of these M2 macrophages towards a M1 phenotype. Interestingly, TGF-β is one of the drivers of c-myc expression in the pro-monocytic cell line U937 [81,82]. Blocking endoglin on macrophages might therefore skew the TGF-β pathway towards smad2/3 signaling, causing the differentiation of macrophages towards an M2 phenotype. This would generate an anti-inflammatory response hampering the anti-tumor responses. Mouse studies on the role of macrophage-specific endoglin expression have been performed. Mice with a floxed endoglin gene were crossed with a myeloid cell-specific Cre (Engfl/fl-LysMCre). Endoglin deletion changed the differentiation and function of macrophages. The authors showed that phagocytic activity by peritoneal macrophages was reduced in the absence of endoglin, leading to sustained infections. Furthermore, altered TGF-β1 expression was found in endoglin-negative peritoneal macrophages, suggesting an M2 phenotype [83]. These studies all suggest that endoglin is important during the polarization to M1 macrophages. 

To study the role of endoglin in a tissue injury model, a study was performed using ENG^+/−^ mice which received kidney irradiation, after which the macrophage function was studied. These results showed impaired IL-1b and IL-6 secretion by macrophages [84] in endoglin heterozygote mice. This again suggests impaired polarization towards M1 macrophages, which are known to secrete IL-1b. As described above, patients with HTT have an increased risk of severe bacterial infections, possibly due to defects of monocyte oxidative burst and phagocytosis [56]. Furthermore, increased levels of Dipeptidyl peptidase-4 (DPP4) were found in patients with HHT, which showed impaired homing towards damaged tissue. An excellent review on mononuclear cells and vascular repair in HHT has been published [85]. In cancer, endoglin is highly expressed by acute myeloid leukemia (AML) subsets. In this study, the authors suggest that endoglin can possibly be used as a potential therapeutic target in AML [86].

Interestingly, all the studies described above indicate that endoglin is involved in the polarization of macrophages. Most studies on endoglin and macrophage function do not discriminate between M1 and M2 phenotypes and the cytokines produced by the macrophages, which hampers exact interpretations of the endoglin function on macrophages. 

### 2.8. Endoglin on Cells of the Adaptive Immune System

Although the majority of endoglin studies have focussed on cells of the innate immune system, more recent work also shows a role for endoglin expression on cells of the adaptive immune system. The adaptive immune system is triggered when a pathogen evades the innate immune system, and consists of B-cells, T-cells, and Natural Killer (NK) cells. Adaptive immunity works closely with the innate immune system. Within adaptive immunity, there is a key role for TGF-β ligands, as recently reviewed in [87]. TGF-β plays an important role in hampering adaptive immunity by inhibiting both the proliferation and effector functions of T-cells. Furthermore, TGF-β induces the differentiation of CD4+ T-cells into T-regulatory cells and TH17 cells, inhibiting the immune response even further. Recent papers describe endoglin expression on lymphocytes, mainly the CD4+ T-cells. Endoglin surface expression seems to be regulated by T-cell receptor activation. The cross-linking of endoglin enhanced CD4+ T-cell proliferation via smad-independent ERK phosphorylation. This study showed that endoglin is expressed by activated CD4+ T-cells and that endoglin is able to counteract the suppressive signal induced by TGF-β [88]. Additionally, more recent unpublished work from our group indicates that a subset of FOXP3-expressing, endoglin-positive regulatory T-cells (Treg) exist. These cells were detected in preclinical mouse models for cancer, as well as in human colorectal tumors. Interestingly, an antibody against endoglin (TRC105/Carotuximab) significantly decreased their number within a mouse MC38 tumor. Although the number of studies is limited, the high abundance and immunosuppressive role of Tregs warrants further investigations of endoglin and regulatory T-cells. Since Tregs play an important role in generating an immunosuppressed environment, targeting them might alleviate this. The role of endoglin on Tregs is currently unknown, but might have to do with counteracting the canonical TGF-βRII/ALK5-dependent TGF-β responses, as shown for macrophages.

### 2.9. Endoglin Expression on Fibroblasts

Fibroblasts are cells of mesenchymal origin and are the main producers of extracellular matrix components. Fibroblasts play an important role in organ development [89], regulating cell differentiation [90] and tissue repair [91]. In healthy tissue, fibroblasts are quiescent and hardly proliferate. Upon tissue injury, a massive expansion of the fibroblast population with an activated phenotype is observed [92]. These activated fibroblasts disappear when the wound is repaired [93]. Under pathologic conditions, this process seems disturbed, leading to sustained fibroblast activation and accumulation, resulting in fibrosis. Activated fibroblasts are characterized by high TGF-β signaling and recent studies show a role for endoglin in this process. Below, we discuss the current knowledge on the endoglin expression of fibroblasts and mesenchymal stem cells, since they show high phenotypic similarities.

### 2.10. Endoglin on Mesenchymal Stem Cells (MSCs) 

Mesenchymal Stem Cells (MSCs) are multipotent cells, which are present in virtually all tissues and organs [94,95]. In vivo, MSCs are thought to be quiescent cells at a perivascular location which are mobilized upon injury in order to promote tissue repair [96]. MSCs suppress overactivation of the immune system, but how they act is still a topic of debate [97]. MSCs are characterized by the expression of CD73, CD90, and endoglin, and the absence of CD45, CD34, CD14, and HLA class II. Endoglin has been reported to be an important MSC marker [98,99], as reflected by the fact that, for clinical applications, MSCs should always express endoglin. The role of endoglin expression in MSCs has not yet been elucidated, but studies have revealed that the absence of endoglin expression on mouse and human MSCs leads to a more differentiated MSC phenotype, with increased osteogenic gene expression [100,101]. Interestingly, when endoglin-negative mouse MSCs were sorted, they were shown to be more efficient in inhibiting T-cell proliferation, compared to their endoglin-expressing counterparts [102]. In addition to healthy MSCs, endoglin expression has also been reported on sarcomas, which are tumors that arise from transformed mesenchymal cells. Endoglin was associated with a worse survival of Ewing sarcoma patients and played a role in a process called vascular mimicry. Moreover, endoglin knockdown in these tumor cells reduces invasiveness and growth [103,104].

### 2.11. Endoglin-Expressing Fibroblasts in Fibrosis

Besides their crucial role in wound healing, the sustained activation and accumulation of fibroblasts can cause tissue damage and fibrosis. Prolonged exposure to inflammatory conditions, induced by tissue-damaging agents, seems to be the underlying cause of most fibrotic diseases [105]. Chemokines, cytokines, and other factors excreted by immune cells lead to the sustained activation of local fibroblasts [106]. One of the key inducers of fibroblast activation is TGF-β [107,108]. TGF-β activates fibroblasts, which in turn start to produce excessive amounts of extracellular matrix (ECM) and proteins involved in the degradation and remodeling of the ECM, like matrix metalloproteinases (MMPs). TGF-β can exert its profibrotic effects directly via TGF-βRII/ALK5-mediated signaling, but there also seems to be a role for endoglin in the profibrotic effects of TGF-β, although this has been less well-established. Several reviews highlight the role of endoglin in liver fibrosis [109,110], myocardial fibrosis [111], and kidney fibrosis [112]. Endoglin expression has been described on profibrotic cells, such as renal fibroblasts [113], myofibroblasts [114], mesangial cells [115], scleroderma fibroblasts [116], and hepatic stellate cells (HSCs) [117]. In liver fibrosis, HSCs upregulate endoglin during trans differentiation, both in vitro and in rat models for liver fibrosis [117]. Furthermore, endoglin overexpression in hepatic stellate cells has been associated with enhanced TGF-β-driven smad1/5/8 phosphorylation and the upregulation of α-smooth muscle actin (α-SMA). Other studies, on the other hand, show that endoglin might also be protective during fibrosis. In a murine model for liver fibrosis, endoglin deficiency enhanced the expression of pro-fibrotic factors such as α-SMA and fibronectin [118]. The authors suggest that endoglin might work protectively by modulating ALK1- versus ALK5-dependent TGF-β signaling. Kapur et al. showed similar findings in a model for heart fibrosis by using an eng+/- mouse model [119]. 

In addition to endoglin expression on stellate cells, several studies have also shown increased levels of sol-eng in circulation during liver fibrosis [120,121,122]. These data suggest that a substantial part of endoglin in fibrotic liver tissues is cleaved and subsequently released into the circulation system. This might be organ-specific, since, in kidney fibrosis (chronic kidney disease and end stage kidney disease), no changes in sol-eng were observed [123]. Although sol-eng was not elevated in patients, in mouse models for kidney fibrosis, unilateral ureteral obstruction (UUO) significantly elevated the mRNA expression of endoglin within the kidney. When the authors investigated if heterozygote mice would develop less fibrosis, they observed no changes in severity of the fibrosis compared to wild-type mice [124]. Another interesting finding in this model was that the overexpression of L-endoglin seemed to increase kidney fibroses after UUO in mice [113], whereas the overexpression of S-endoglin seemed to reduce kidney fibrosis and inflammation [125]. Although overexpression was not fibroblast-specific, the authors showed that L-endoglin increased both the smad1/5/8 and smad2/3 pathways, while S-endoglin showed the decreased phosphorylation of both smad1/5/8 and smad2/3 pathways. These data indicate that the effects are dependent on the cytoplasmic domain. 

Finally, endoglin expression has also been studied in cardiac fibrosis [111]. Endoglin expression on cardiac fibroblasts was highly upregulated upon TGF-β1 stimulation [126] and mediated the profibrotic effects of angiotensin II on cardiac fibroblasts [127,128]. Furthermore, sol-eng limits TGF-β1 signaling in cardiac fibroblasts. Interestingly, treatment with sol-eng limited cardiac fibrosis in an in vivo model for heart failure [119].

Taken together, there is no consensus about the pro- or anti-fibrotic role of endoglin. The role of endoglin might be cell- and tissue type-specific. Interestingly, many studies show that endoglin might restore the balance between the smad2/3 pathway and smad1/5/8 pathway, balancing the TGF-β induced signaling.

### 2.12. Endoglin Expression in Cancer-Associated Fibroblasts (CAFs) 

In various solid tumors, a high accumulation of fibroblasts with an activated phenotype, called CAFs, is observed and their abundance seems to predict patient survival [129]. CAFs can stimulate cancer progression via stimulating the growth and secretion of pro-invasive, pro-metastatic, and pro-angiogenic factors. The origin of CAFs is most probably heterogeneous and composed of activated local fibroblasts and bone marrow cells [130,131,132,133], or results from epithelial to mesenchymal transition (EMT) [134] and endothelial to mesenchymal transition (EndMT) [135,136]. These various sources might also lead to various CAF subsets, all with distinct roles in immune regulation, tumor progression, and metastasis [137,138]. Similar to its role in fibrosis, TGF-β is a main driver of CAF activation, mostly via the ALK5 signaling pathway. In addition, recent studies show an additional role for endoglin. The striking phenotypical resemblance between CAFs and MSCs makes it in hard to distinguish CAFs from MSCs in tumors, so both subsets are described to express endoglin. 

Romero et al. were the first to describe endoglin on CAFs in prostate cancer [139]. They showed, in Transgenic Adenocarcinoma of the Mouse Prostate (TRAMP) mice on an endoglin heterozygote background, that tumors are less fibrotic and less prone to form metastasis. Furthermore, endoglin-expressing CAFS were able to promote neovascularization and tumor growth, suggesting that endoglin on CAFs in prostate tumor mediates metastasis and tumor growth. Our study on colorectal cancer (CRC) shows that endoglin-expressing α-SMA+ CAFs at the invasive front of CRC, are related to metastasis-free survival. Furthermore, when we targeted endoglin on these CAFs in a mouse model for experimental liver metastasis, a reduction in the number of metastases was detected [140], in line with earlier results reported for prostate cancer. Although the number of studies describing endoglin on CAFs is still limited, there seems to be a tumor-promoting role for endoglin-expressing CAFs. 

Single-cell RNA sequencing studies on breast cancer have identified a subpopulation of so-called vascular CAFs (vCAFs), which are characterized by their expression of endoglin [141]. In pancreatic cancer, endoglin is only expressed in cluster 12, which has been defined as an endothelial (non-CAF) cell cluster [142]. In contrast to that, our unpublished data show strong endoglin expression on CAFs in human pancreatic ductal adenocarcinoma (PDAC). In gastric and breast cancer, a strong association between endoglin-expressing CAFs/MSCs and a poor prognosis was reported [143,144]. 

These studies suggest that there seems to be a pro-tumerogenic/pro-metastatic role for endoglin expression on CAFs, potentially via regulating/balancing ALK1 versus ALK5 pathways. The identification of CAF subsets, using multiomics data, is rapidly increasing and should reveal the potential for endoglin targeting on these CAF subsets.

## 3. Targeting Endoglin in Diseases

Because of the high endothelial endoglin expression, therapies targeting endoglin have been evaluated [9,11,145], mostly focussed on its endothelial expression. With increasing knowledge on endoglin expression beyond the endothelium, it might be that endoglin targeting directly targets other cell types. In cancer, TRC105 has been clinically tested and although encouraging results have been published [146,147,148,149,150], a recent phase-III trail in angiosarcomas did not show clinical efficacy in the interim analysis. 

**Figure 2:**
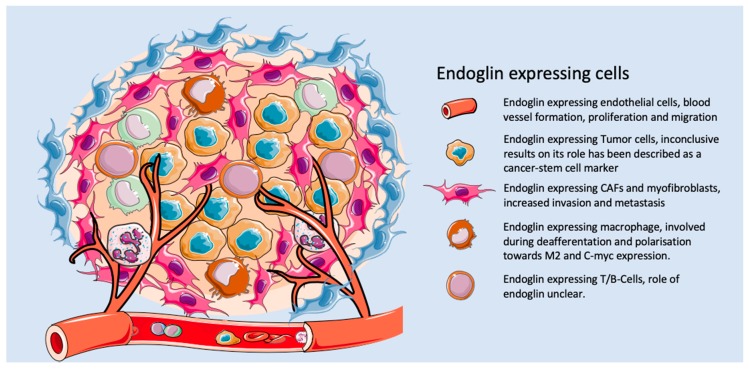
The role of endoglin on different cell types within the tumor microenvironment.

Based on the data above, TRC105 might not only target endothelial cells, but also other cells in the tumor microenvironment (Figure 2). This has been shown in pre-clinical models for breast cancer, where a decrease in the amount of α-SMA-positive cells was reported by our group upon TRC105 treatment. Interestingly, in the reported clinical studies with TRC105, like in a phase-II study for advanced metastatic urothelial carcinoma, a decrease in circulating Tregs was observed [151]. Our own unpublished data might provide an explanation for this phenomenon, since we have detected a subset of endoglin-expressing Tregs in CRC, which can be depleted using TRC105. Further validation of these findings should show if this is an additional target of endoglin therapy.

Next to regulatory T-cells, endoglin is highly expressed on some tumor cells directly targeting endoglin-expressing tumor cells, which might induce a direct anti-tumor response. In urothelial carcinoma patients treated with TRC105, a decreased number of circulating tumor cells were observed [151], although the authors did not show that this is a direct effect of the targeting of circulating tumor cells (CTCs) by TRC105. This also correlates with data demonstrating that endoglin targeting inhibits metastatic spread in pre-clinical models for breast [152] and colorectal cancer [140]. These first data open up many new possibilities to look back at valuable data obtained from clinical studies involving TRC105 and its effects on non-endothelial cells.

## 4. Concluding Remarks

Originally identified on endothelial cells, more recent work has shown additional and not yet defined roles for endoglin on other cell types. Although the numbers of studies investigating endoglin on non-endothelial cells has increased, much is still unknown. Endoglin expression seems to be strongly upregulated in a multitude of cells upon in vitro cell culture, possibly due to the activation status or stress of the cells, hampering thorough mechanistic studies. Next to that, there seem to be opposing roles for endoglin in different tissues/diseases. It is clear that multiple cells can express endoglin, mainly in a TGF-β environment, such as cancer, in which both CAFs and some immune cells express endoglin. The exact role of endoglin expression beyond the endothelium is still unclear, but nevertheless represents an exciting new area of research.
